# Association of Gastric Sarcina With Malignant Pyloric Stenosis

**DOI:** 10.14309/crj.0000000000001593

**Published:** 2025-01-29

**Authors:** Shivangini Duggal, Keith Garrison, Samantha Meza-Rodriguez, Ben Williams, Ioannis Konstantinidis, Marc J. Zuckerman, Sherif E. Elhanafi

**Affiliations:** 1 Department of Internal Medicine, Texas Tech University Health Sciences Center El Paso, El Paso, TX; 2 Division of Gastroenterology, Texas Tech University Health Sciences Center El Paso, El Paso, TX; 3 Department of Pathology, Texas Tech University Health Sciences Center El Paso, El Paso, TX; 4 Division of Surgical Oncology, Texas Tech University Health Sciences Center El Paso, El Paso, TX

**Keywords:** sarcinia ventriculi, gastric outlet obstruction, signet ring cell carcinoma

## Abstract

*Sarcina ventriculi* (SV) is a Gram-positive cocci that thrives in the acidic stomach environment and may cause gastrointestinal symptoms. A 65-year-old woman with a history of *Helicobacter pylori* gastritis and diabetes presented with abdominal pain, vomiting, diarrhea, and weight loss. Initial esophagogastroduodenoscopy revealed pyloric stenosis with thickened prepyloric gastric folds, and endoscopic biopsy revealed SV without malignancy. Owing to persistent symptoms, endoscopic ultrasound was done with repeat biopsies and was nondiagnostic. Subsequently, a robotic gastrojejunostomy was done due to persistent gastric outlet obstruction symptoms. Surgical specimens revealed signet ring cell carcinoma. This case highlights the importance of suspecting underlying malignancy in patients with SV and the necessity of comprehensive diagnostic evaluation when endoscopic findings are inconclusive.

## INTRODUCTION

*Sarcina ventriculi* (SV) (or *Clostridium ventriculi*) is a Gram-positive coccus that grows in acidic environments and uses carbohydrates as its sole energy source for fermentative metabolism.^1^ Its morphological characteristics include nearly spherical shape, individual sizes ranging from 1.8 to 3 µm, refractory nature, basophilic staining with hematoxylin-eosin, flattened cell walls, and presence of extracellular cellulose.^2^ It typically occurs in distinctive tetrads or packets of 8 or more cells, resulting from division in perpendicular planes.^3^

It is a commensal organism found in soil and human feces which has been isolated from the gastrointestinal, respiratory, and urinary tracts in humans. The bacterium is hypothesized to cause damage to the gastric mucosa due to acetaldehyde accumulation. Several reports have shown that the bacterium is associated with delayed gastric emptying.^4,5^ Underlying conditions that promote gastric retention, such as diabetic gastroparesis, pyloric stenosis, and gastric surgery, have been known to promote its overgrowth.^6^ SV has been associated with dyspepsia, abdominal pain, gastric ulcers, and, in rare cases, emphysematous gastritis, which may lead to gastric perforation.^7,8^

We present a case of a 65-year-old patient with upper gastrointestinal symptoms who was found to have a gastric malignancy associated with SV found on endoscopic biopsy.

## CASE REPORT

**Figure 1.(:**
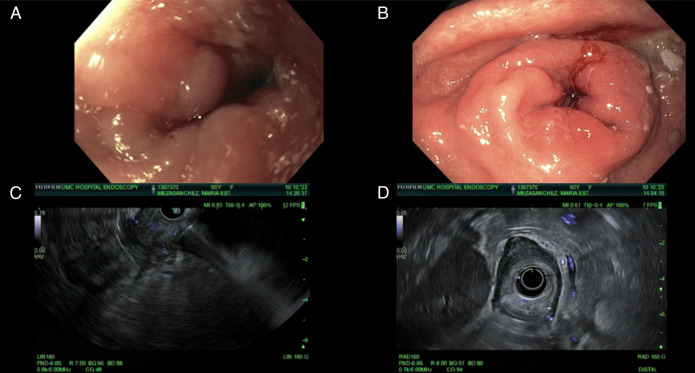
A and B) Endoscopic findings demonstrating severe stenosis in the prepyloric region of the stomach. (C and D) Demonstrate endoscopic ultrasound findings of localized wall thickening in the prepyloric region of the stomach.

**Figure 2.(:**
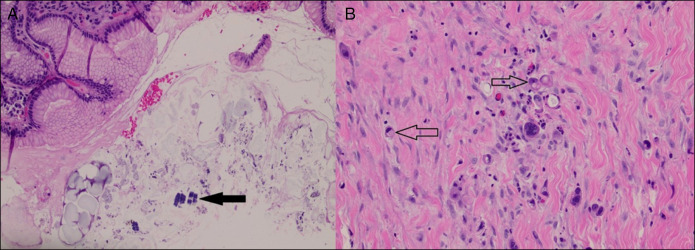
A) 200×, demonstrating a H&E slide showing the basophilic color and cocci tetrad arrangement characteristic of *Sarcina ventriculi* (black arrow). (B) 200× magnification, demonstrating a H&E slide showing signet ring cell carcinoma features (transparent arrow). H&E, hematoxylin and eosin.

A 65-year-old woman with a medical history of untreated *Helicobacter pylori* gastritis (found on gastric biopsy), diabetes mellitus type 2, and hypertension was presented with sudden onset abdominal pain for 3 days accompanied by episodes of vomiting, diarrhea, and weight loss of 12 kg over 1 month. The patient underwent a computed tomography of the abdomen and pelvis on admission which showed gastric distention indicating gastroparesis or gastric outlet obstruction (GOO). Owing to these findings, the patient underwent esophagogastroduodenoscopy (EGD), which revealed severe pyloric stenosis with circumferential thickening of the prepyloric gastric folds suspicious for gastric mass. Cold forceps biopsies were obtained and, although negative for malignancy, demonstrated intestinal metaplasia with staining revealing the presence of *Candida* and SV. The examined duodenum was normal on all EGDs. The patient subsequently underwent a positron emission tomography scan which showed no metabolic activity in the distal esophagus or stomach. She was later readmitted due to persistent complaints of abdominal pain, vomiting, and diarrhea. A repeat EGD and endoscopic ultrasound (EUS) revealed a localized wall thickening in the prepyloric region mainly within the submucosa (Layer 3) and muscularis propria (Layer 4) (Figure 1), with the gastric wall measuring up to 11 mm in thickness. A fine-needle biopsy was performed at the prepyloric region. Using a 22-gauge Acquire biopsy needle, 3 transgastric passes were made in the prepyloric and pyloric region, yielding a visible core of tissue for analysis. Pyloric stenosis was dilated using through the scope dilator up to 12 mm. The results of US-guided biopsies were inconclusive and negative for malignancy. Owing to ongoing concern for underlying malignancy despite multiple negative biopsies, multidisciplinary discussions between surgical oncology and gastroenterology teams led to the decision to proceed with a robotic exploration and gastrojejunostomy, as treatment for her significant GOO. During the procedure, multiple peritoneal deposits were visualized in the right and left upper quadrant, omentum, and on the posterior surface of the stomach. Stomach wall biopsy from the surgery was negative for malignancy but consistent with *Candida* infection. Biopsy of peritoneal nodules revealed several foci of metastatic signet ring cell carcinoma (SRC), positive for pan-keratin and CDX2 immunostains, some were even positive for mucicarmine stain and negative for HER2/neu (Figure 2). Although the stomach wall biopsy from the robotic exploration did not demonstrate malignancy, the peritoneal metastasis findings were consistent with a primary gastric origin, given the morphologic and immunohistochemical profiles. This evidence led to the diagnosis of stage IV metastatic gastric SRC. The patient was subsequently started on palliative chemotherapy with leucovorin, fluorouracil, and oxaliplatin.

## DISCUSSION

Our case highlights the potential association of the rare bacterial organism SV with gastric malignancy.

SV was first observed and documented by John Goodsir in 1842, in the stomach contents of a patient experiencing vomiting. SV was renamed from *Clostridium ventriculi* because the genus *Sarcina* was established earlier (1842) than *Clostridium* (1880), giving it nomenclatural priority. Although both genera share some genetic similarities (e.g., 16S rRNA), *Sarcina* is distinct in its morphology and ecological niche, justifying the separation into different genera.^9^ *Sarcina* infections have a female predominance with a male:female ratio of 1:2 and is common in the diabetic population.^10^ A case isolating *Sarcina* species in a middle-aged woman with GOO due to edematous antral folds has been described.^10^ Although the pathogenicity of SV in humans remains unclear, the aforementioned case report showed improvement of symptoms with ciprofloxacin and metronidazole. Some authors suggest that SV directly affects a healthy stomach by depositing acetaldehyde and ethanol, formed during carbohydrate fermentation, which can cause stomach and duodenal damage similar to that seen with severe alcohol intake.^11^ In addition, many patients experience abdominal distention due to carbon dioxide produced by glucose fermentation and pyruvate metabolism.^12^ Conversely, it has been proposed that SV thrives in the human gut due to delayed gastric emptying from conditions such as diabetic gastroparesis, gastric reconstructive surgery, scarring, and pyloric stenosis. It likely exerts its effects through a combination of preexisting mucosal damage and delayed gastric emptying, which increases the availability of nutrient substrates.^13^ Their fermentative carbohydrate metabolism may explain the emphysematous damage often associated with this infection.^14^

*Sarcina ventriculi* association with gastric malignancy has been sporadically described in the literature. Two women in their 50s, presenting with symptoms of GOO, were found to have SV in biopsies from the pylorus and were ultimately diagnosed with gastric adenocarcinoma.^5^ *Sarcina* species have been linked to gastric ulcer, emphysematous gastritis, and peritonitis due to gastric perforation, often associated with GOO and ulcer formation.^5,15^ The presence of these organisms may indicate delayed gastric emptying and its complications, underscoring the need to investigate potential underlying conditions, such as occult malignancies, as seen in this case. Malignant mechanical GOO can occur with cancers in the pylorobulbar area, the antropyloric zone, and the descending duodenum. Distal gastric cancer (GC) is the most common malignancy, accounting for up to 35% of cases of GOO.^16^ A literature review of 55 articles reported 65 cases of *Sarcina ventriculi*-related gastritis. The median age at presentation was 51 years, and 49% of the patients presented with abdominal pain. EGD was performed in 79% cases, and the majority (46%) cases showed a gastric ulcer.^3^ Diagnosis is established through histopathological examination of endoscopic biopsy in cases of gastric *Sarcina* infection. In the era of increased utilization of endoscopic-guided gastrojejunostomy with lumen-apposing metal stents for GOO, this approach might have initially been considered in this patient. However, given the high pretest probability of malignancy and the inconclusive nature of multiple endoscopic biopsies, an exploratory laparoscopy was ultimately deemed necessary for both diagnostic and therapeutic purposes. The decision for a robotic exploration allowed direct visualization of the peritoneal surfaces and identification of metastatic peritoneal deposits, confirming the diagnosis of metastatic SRC.

The relationship between *H. pylori* infection and SRC remains complex and somewhat controversial. *H. pylori* is recognized as a major risk factor of GC, especially nonsignet ring adenocarcinoma. However, its role in SRC is less definitive.^17^ In a Japanese study, it was found that while the overall incidence of GC declined with reduced *H. pylori* infection rates, SRC was less frequently associated with the marked mucosal atrophy typically induced by *H. pylori*.^18^ Although some studies report high rates of *H. pylori* seropositivity in diffuse-type gastric cancers, including SRC, other research suggests that SRC may be more influenced by genetic or molecular factors than by *H. pylori*-associated inflammation and atrophy, suggesting that *H. pylori* may act more as a cofactor than a primary cause in SRC pathogenesis.^19,20^ Linkage analysis has implicated mutations in CDH1 (E-cadherin) in approximately 25% of families with an autosomal-dominant predisposition to diffuse-type GC, highlighting a potential genetic contribution.^21^ In addition, *H. pylori* infection has been linked to specific DNA methylation alterations in the gastric mucosa, including CDH1 gene methylation in sporadic diffuse-type GC associated with *H. pylori*, which may reverse on *H. pylori* eradication.^22^ In our case, biopsies from the gastric antrum and body were positive for *H. pylori* on Giemsa stain (no testing was performed for Cag PAI strain) a year ago before this presentation; however, all subsequent gastric biopsies were negative for *H. pylori*.

The medical treatment of SV infection typically involves metronidazole alone or in combination with ciprofloxacin and/or a proton-pump inhibitor.^23^ There is no widely agreed-upon standard treatment regimen or duration, and the effectiveness of different regimens remains unclear due to lack of follow-up biopsies.^24^ Debate persists on whether antibiotics should be universally administered, with some patients achieving eradication without antibiotic therapy. Surgical intervention is required in emergent cases of gastrointestinal perforation or when addressing underlying conditions that promote *Sarcina* growth, such as delayed gastric emptying or GOO.^25^ Our patient never received treatment of *Sarcina* infection as the underlying malignant obstruction was treated with palliative chemotherapy.

In conclusion, SV is a rare Gram-positive coccus, associated with GOO due to gastroparesis or malignancy. The presence of SV on gastric biopsy should lead clinicians to investigate underlying disorders, which may predispose its growth. This case highlights the importance of determining the cause of GOO and seeking surgical evaluation if endoscopic evaluations are inconclusive.

## DISCLOSURES

Author contributions: This paper was conceptualized by S. Duggal, K. Garrison, and S. Meza-Rodriguez. The investigation and review of relevant data and articles were done by S. Duggal, K. Garrison, and S. Meza-Rodriguez. Case analysis was done by S. Duggal, S. Meza-Rodriguez, B. Williams, and K. Garrison. The original draft was written by S. Duggal, S. Meza-Rodriguez, and K. Garrison. Review and editing were done by M. Zuckerman, I. Konstantinidis, and SE Elhanafi. S. Duggal is the article guarantor. All authors discussed the findings described in the case and approved the final manuscript.

Financial disclosure: None to report.

Previous presentation: This case report was presented as an abstract at the Digestive Diseases Week; May 2024; Washington, DC.

Informed consent was obtained for this case report.
